# An unusual case of left ventricular aneurysm in duchenne muscular dystrophy

**DOI:** 10.1186/1476-7120-8-49

**Published:** 2010-11-14

**Authors:** Xiaozhou Du, Matthew Zeglinski, Nasir Shaikh, Davinder S Jassal

**Affiliations:** 1 Institute of Cardiovascular Sciences, St. Boniface General Hospital, University of Manitoba, Winnipeg, Manitoba, Canada; 2 Section of Cardiology, Department of Internal Medicine, University of Manitoba, Winnipeg, Manitoba, Canada; 3 Department of Radiology, University of Manitoba, Winnipeg, Manitoba, Canada

## Abstract

Duchenne muscular dystrophy (DMD) leads to structural heart disease, including dilated cardiomyopathy, in 90% of patients >18 years of age. Despite the ubiquity of cardiomyopathy associated with DMD, ventricular aneurysms in these patients have rarely been reported. We present a case of a basal inferoposterior aneurysm of the left ventricle in a 23-year-old male patient with DMD.

## Case Report

**Figure 1:**
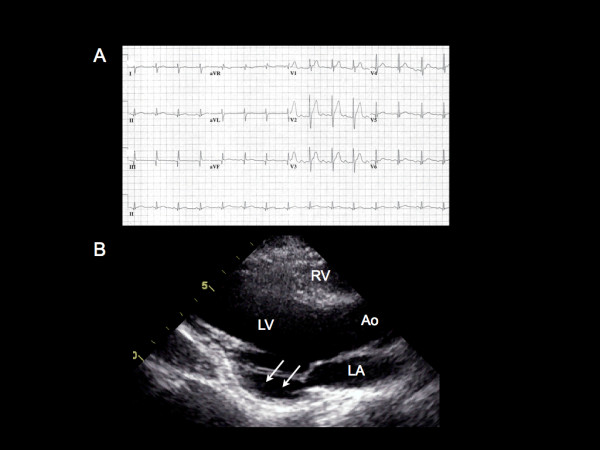
**12 Lead EKG and transthoracic echocardiography.A**. The ECG demonstrates right axis deviation, prominent R waves in V1, and Q waves in the inferior and lateral leads (II, III, AVF, and V4-6). **B**. A parasternal long axis view on transthoracic echocardiography demonstrating a true aneurysm (15 mm × 27 mm) of the basal inferoposterior wall of the LV (arrow). RV, right ventricle; LV, left ventricle; Ao, aorta; LA, left atrium.

Mr. W.D. is a 23-year-old male patient with DMD. He was referred for evaluation of cardiac abnormalities due to underlying DMD. He denied chest discomfort, shortness of breath, palpitations nor syncope. On physical examination, his blood pressure was 100/60 mm Hg with a heart rate of 72 beats per minute. The jugular venous pressure was within normal limits. There were normal cardiac heart sounds with no murmurs and no evidence of pedal edema. Twelve lead electrocardiography demonstrated right bundle branch block with Q waves in the inferolateral leads (Figure 1A). Transthoracic echocardiography (TTE) demonstrated a mildly dilated LV with an inferoposterior wall aneurysm of the left ventricle (LV) (Figure 1B). The overall left ventricular ejection fraction (LVEF) was mildly reduced at 40-45%. Due to the low risk of rupture of a true LV aneurysm, the patient was treated conservatively.

## Discussion

Duchenne muscular dystrophy (DMD) is an x-linked dystrophinopathy that occurs in 1/3600 to 1/6000 males [1]. Approximately 1/3 of DMD is caused by de novo mutations, while 2/3 are inherited [2]. DMD presents early at the age of 2 to 3, with patients often demonstrating difficulty mobilizing, characteristic waddling gait and lumbar lordosis [1-3]. The diagnosis of DMD is often made by 5 years of age when the patients' physical capacities diverge markedly from their peers [1]. The mean age for loss of ambulation is 10 years [3]. The diagnosis of DMD is often made with genetic investigations and muscle biopsy, along with the family history and the clinical presentation. Characteristic blood biomarkers such as elevated serum creatinine kinase (CK) and myoglobin are indicative, but not diagnostic [2].

Cardiomyopathies are noted in DMD patients as young as 10 years of age [2]. Approximately 90% of DMD patients suffer from an underlying cardiomyopathy, with dilated cardiomyopathy constituting majority of cases [2]. Despite the high prevalence of cardiac disease, cardiac symptoms were reported in only 57% of DMD patients over 18 years of age, which suggests that many patients remain asymptomatic, likely due to their low physical capability [2]. Severe dilated cardiomyopathy may lead to heart failure, which accounts for 20% of mortalities in DMD patients [2,3].

Cardiac involvement in DMD is caused by the progressive loss of cardiomyocytes, which is replaced with connective tissue and lipid infiltrates, creating wall motion abnormalities, reduced LVEF and conduction anomalies [2]. The loss of cardiomyocytes also results in higher wall stress, coupled with reduced physiological adaptability of the dystrophin-deficient myocardium, leading to LV dilation and systolic dysfunction [3].

A screening electrocardiography (ECG) is recommended in all DMD patients [1]. The most common ECG abnormality is sinus tachycardia, with a prevalence of approximately 90% [2]. Other ECG abnormalities include premature atrial and ventricular contractions, conduction blocks, atrial flutter or atrial fibrillation [2]. As the posterior wall of the LV is often most affected in DMD, patients typically present with tall R waves in V1 and deep Q waves in the inferolateral leads, as demonstrated in our patient [2]. Other common ECG features include ST depression, prolonged QT interval and increased QT dispersion [2].

Due to the prevalence and severity of cardiac disease in DMD patients, recent guidelines recommend TTE at the age of diagnosis, followed by every 2 years until 10 years of age, and annually thereafter [1]. Echocardiographic evidence of structural heart disease in DMD patients include LV hypertrophy, regional wall motion abnormalities, dilation of the cardiac chambers, valvular abnormalities, and LV systolic dysfunction [2]. Furthermore, LV wall motion abnormalities progress in a set sequence in DMD patients, initially involving the posterior wall and the apex, followed by the interventricular septum and finally the anterior wall [4].

Despite the pervasiveness of structural heart disease in DMD patients, an isolated left ventricular aneurysm (LVA) is a rare occurrence. Ventricular aneurysm can be visualized on TTE as a dyskinetic or akinetic evagination of the ventricular wall [5]. Transthoracic echocardiography has a 93% sensitivity and 94% specificity for identifying LVA [5].

True LVA commonly result from myocardial injury, leading to thinning and fibrosis of all three layers of the myocardium, with subsequent remodelling of the ventricle [5]. As the incidence of rupture is low, true LVA's are managed conservatively [5,6]. Indications for surgical treatment of LVA include refractory heart failure despite optimal medical therapy, intractable angina, systemic embolization, and/or incessant ventricular arrhythmias [6]. Our case report serves as the first illustration of an asymptomatic inferoposterior aneurysm of the LV in a DMD patient in the English literature that was managed conservatively.

## Conclusion

Despite the ubiquity of cardiomyopathy associated with DMD, ventricular aneurysms may be incidentally detected on routine non-invasive cardiac imaging.

## List of Abbreviations

DMD: Duchenne muscular dystrophy; ECG: electrocardiography; LV: left ventricle; LVA: left ventricular aneurysm; LVEF: left ventricular ejection fraction; TTE: transthoracic echocardiography.

## Consent

Written informed consent was obtained from the patient for publication of this case report and accompanying images. A copy of the written consent is available for review by the Editor-in-Chief of this journal.

## Competing interests

The authors declare that they have no competing interests.

## Authors' contributions

XD, MZ, NS, and DJ contributed to the writing of the manuscript. All authors read and approved the final manuscript.

## Note

All work was preformed at the St. Boniface General Hospital, University of Manitoba 409 Tache Avenue, Winnipeg, Manitoba, Canada, R2H 2A6
